# Preparation of Sulfur of High Purity

**DOI:** 10.6028/jres.064A.036

**Published:** 1960-08-01

**Authors:** Thomas J. Murphy, W. Stanley Clabaugh, Raleigh Gilchrist

## Abstract

A method is described for producing sulfur that contains less than 1.3×10^−5^ mole fraction of liquid-soluble, solid-insoluble impurities as determined by the freezing point depression. This corresponds to a purity of 99.999 mole percent. Many of the impurities, including organic matter, are removed by oxidation with sulfuric and nitric acids. The nonvolatile impurities are removed on distilling the sulfur. The residual sulfuric acid is removed by a special extraction with distilled water.

Methods are described for determining small amounts of the following impurities: Selenium, tellurium, arsenic, iron, carbon, sulfuric acid, and residue after ignition.

## 1. Introduction

The boiling point of sulfur is one of the fixed points on the International Temperature Scale. The sulfur used for determining this temperature must be as pure as possible and, expecially, free of impurities that would affect its boiling point. Analysis of sulfur to be used by Harold F. Stimson of this Bureau in a study of the factors affecting the precise determination of this fixed temperature point showed that the main impurity was organic matter. Inspection of several other samples of so-called pure sulfur revealed that they all contained various amounts of organic impurities.

Several attempts to purify sulfur by methods which are described in the literature always resulted in sulfur that contained appreciable amounts of organic impurities. This was especially true for sulfur that had been recrystallized from carbon disulfide. When roll sulfur containing 140 parts of carbon per million was purified by the method of Von Wartenburg [1],^1^ which consists of heating the sulfur at 200° C for 48 hr in an atmosphere of nitrogen and then distilling it, the sulfur was found to contain organic matter to the extent of 53 parts of carbon per million parts of sulfur.

Some of the same sulfur purified by the method of James [2], which consists of agitating the sulfur at a temperature above its melting point with about 2 percent of its weight of concentrated sulfuric acid for a period ranging from 15 min to 2 hr and allowing the mixture to separate into two layers, was found to contain 60 parts of carbon per million of sulfur.

The method of Bacon and Fanelli [3], in which sulfur is boiled with 1 percent of magnesium oxide and then decanted, was not tried because Yeisen [4] reported that magnesium salts are somewhat soluble in sulfur and these salts might affect the boiling point of sulfur.

This paper describes a reliable method for purifying sulfur and the analytical methods used to establish its purity.

## 2. Method of Purification Adopted

The starting material was commercial roll sulfur which was found by the analytical methods described later to contain 76 parts of nonvolatile matter, 11 parts of iron, 140 parts of carbon, and less than 1 part of selenium, 1 part of tellurium, and 0.5 part of arsenic per million parts of sulfur. In addition to the above impurities, bits of wood and other foreign matter were present.

The sulfur was freed of this obvious foreign matter by filtering the melted sulfur through a glass filtering funnel of coarse porosity. About 2.5 kg of the filtered sulfur was transferred to a 2-liter, round-bottom Pyrex flask that was equipped with two necks, each having standard taper 24/40 ground-glass joints. About 300 ml of concentrated sulfuric acid was added to the flask and the mixture was heated until the sulfur melted. A motor-driven glass stirrer was inserted through one of the necks. The mixture was heated to 150° C and continuously stirred. Concentrated nitric acid was added in about 2-ml portions at intervals of about 10 to 15 min for a period of 6 hr.

It should be pointed out that the reaction of hot sulfur with sulfuric and nitric acids produces a bluish-black color in the acid layer. This color masks the color produced by the action of hot sulfuric acid on the organic material, making it difficult to tell when all of the organic impurity is removed. However, with all the various samples of sulfur tried, the treatment described above sufficed to reduce the amount of organic impurity to a very low concentration.

The acid-sulfur mixture was allowed to cool to room temperature, then the acid was poured off, and the sulfur was rinsed several times with distilled water. Since some of the acid remains entrapped in the sulfur, the sulfur was remelted and allowed to cool, and again rinsed several times with distilled water. This procedure of remelting, cooling, and rinsing was repeated four or five times to remove most of the sulfuric and nitric acids.

An air-cooled reflux tube, about 40 cm long, was then attached to one of the necks, and a gas delivery tube sealed through a standard taper joint was placed in the other neck. The lower end of the delivery tube was about 1 in. above the bottom of the flask. The sulfur was then boiled at such a rate that its vapors condensed within a few inches of the top of the reflux tube. A current of helium or water-pumped nitrogen was continually passed through the boiling sulfur. This stream of gas aided in sweeping out the vapors of water, nitric, and sulfuric acids. This refluxing was continued for about 4 hr. The sulfur was cooled and the reflux tube was replaced with a bent air-cooled condenser. The sulfur was then distilled. The first 100-ml portion was discarded and a portion of about 100 ml was allowed to remain in the distilling flask.

To further reduce the acid and water content of the sulfur, the distilled sulfur was melted and transferred to 400-ml glass cylindrical ampoules. Each ampoule was filled to slightly less than one-half of its volume and then placed on its side until the sulfur solidified. Solidifying the sulfur with the ampoules in this position was necessary to prevent the ampoules from breaking when the sulfur was remelted for subsequent treatment. The glass ampoules were constructed of 50-mm tubing and were about 20 cm long; the necks were about 15 cm long and made of 10-mm glass tubing.

About 80 ml of water was added to each 400-ml ampoule, which contained about 200 ml of sulfur. The air remaining in the ampoule was displaced with water-pumped nitrogen, which prevented the formation of sulfuric acid from the reaction of the oxygen present with the melted sulfur. The ampoules were then sealed and placed on their sides in an oven heated to 125° C. After the sulfur had melted, each ampoule was shaken to extract residual sulfuric acid into the water. The ampoules were again placed on their sides to cool. After cooling, the tips of the ampoules were broken open, and the water was poured off and titrated with 0.02 *N* sodium hydroxide solution to determine the sulfuric acid content. Eighty milliliters of water was again added to each ampoule and the process was repeated until no change in acid was noted. Three extractions were sufficient in most cases to reduce the sulfuric acid to less than 0.0002 percent.

**Figure 1:**
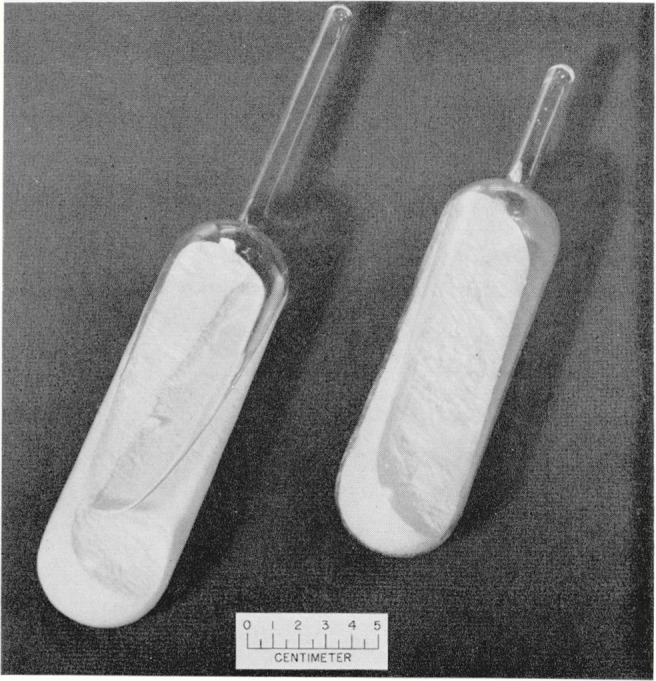
Photograph of a sealed ampoule containing sulfur and water (left) and a sealed ampoule containing the purified sulfur (right).

This extraction process removed the excess acid, but the sulfur contained entrapped water in addition to any water that might have been dissolved in it. To remove the water remaining in the ampoule after the last extraction the ampoule was sealed to a vacuum system. A trap immersed in a dry ice bath was sealed between the ampoule and the vacuum pump. The sulfur was slowly melted while the system was being evacuated and the pumping was continued until the pressure in the system was reduced to 10 mm of mercury. The pumping was interrupted and water-pumped nitrogen was admitted to a pressure of 100 mm of mercury, and the system was again evacuated to a pressure of 10 mm of mercury. The sulfur was maintained in the molten state throughout these operations. Water-pumped nitrogen was then admitted until the pressure was 1 atm. The ampoule was sealed off and placed on its side for the sulfur to solidify. Figure 1 shows a photograph of a sealed ampoule containing sulfur and water and a sealed ampoule containing the final purified product.

## 3. Estimation of Absolute Purity

The thermodynamic properties of this purified sulfur were measured by West [5] by means of an adiabatic calorimeter. The mole-fraction of liquid-soluble, solid-insoluble impurity was calculated to be 1.3×10^−5^ from the freezing point depression. This corresponds to a purity of 99.999 mole percent of sulfur.

## 4. Methods for Determining Impurities in the Sulphur

### 4.1. Separation and Determination of Selenium and Tellurium

The selenium and tellurium were separated from the sulfur by dissolving the sulfur in cold liquid bromine and extracting the selenium and tellurium bromides in cold water. The selenium and tellurium were precipitated as the elements [6]. The elements were dissolved in hydrobromic acid and separated by distillation.

#### Recommended Procedure

Dissolve 50 g of sulfur in 55 ml of chilled bromine. Cool the solution in an ice bath, transfer it to a separatory funnel, and add 10 ml of cold distilled water. Shake the mixture for 1 min, or until the solution begins to get warm. Draw off the lower layer containing the sulfur bromide, and pass the aqueous layer through a wetted filter paper to catch any globules of sulfur bromide. Repeat the extraction of the sulfur bromides with cold distilled water several times, combine the water extracts, and dilute the resulting solution to a volume of 100 ml.

Pass a stream of sulfur dioxide into the aqueous extract, to reduce the excess bromine to bromide. Add 0.5 g of hydrazine sulfate and allow the solution to stand overnight to precipitate selenium and tellurium. Filter the solution through a glass micro-filtering crucible of fine porosity and wash the precipitate with distilled water. Dissolve the mixed precipitate in about 30 ml of colorless concentrated hydrobromic acid to which a drop of bromine is added. Dilute the solution so obtained to a volume of 50 ml with concentrated hydrobromic acid, and transfer it to a 100-ml distilling flask. Distill 25 ml of this solution into 25 ml of water, and destroy the excess bromine in the distillate with sulfur dioxide, as described above. To this solution add 0.5 g of hydroxylamine hydrochloride and 2 ml of 0.05-percent solution of gum arabic, and place the solution on the steam bath for 1 hr. Cool the solution and compare its red turbidity with control solutions containing known amounts of selenium. Selenium in an amount as small as 0.05 mg can easily be determined; this corresponds to 1 ppm in the sulfur.

Pour the solution, which remains in the distilling flask after the elimination of selenium, into a beaker and compare its yellow color with control solutions containing known amounts of tellurium in equal volumes of concentrated hydrobromic acid. Tellurium in an amount as small as 0.05 mg can easily be determined; this corresponds to 1 ppm in the sulfur.

### 4.2. Determination of Arsenic

Arsenic was determined by the standard Gutzeit method [7].

#### Recommended Procedure

Dissolve 3 g of sulfur in 3.5 ml of liquid bromine. Add 75 ml of diluted nitric acid (1+9),^2^ and heat the resulting solution to boiling. Continue to boil the solution until reaction ceases. Concentrate the solution as far as possible on the steam bath, add a small amount of bromine to dissolve any free sulfur, and add 5 ml of diluted nitric acid (1 + 1). This treatment with bromine and nitric acid converts the sulfur to sulfuric acid. The nitric acid also oxidizes any arsenic present to the quinquevalent state so that it will not be volatilized in the subsequent evaporations. Heat the solution to boiling and finally evaporate it until fumes of sulfuric acid are evolved.

Determine the arsenic content by the standard Gutzeit method [7], comparing the stain produced with those of known amounts of arsenic. Arsenic in an amount as small as 0.002 mg can be determined; this corresponds to 0.7 ppm in the sulfur.

### 4.3. Determination of Residue After Ignition

#### Recommended Procedure

Burn 50 g of the sulfur in a tared dish, in a well-ventilated hood, ignite the residue at a temperature of 550° to 650° C, cool it in a desiccator, and weigh it.

### 4.4. Determination of Iron

#### Recommended Procedure

Add 10 ml of diluted hydrochloric acid (1 + 1) to the residue obtained in section 4.3. Digest the mixture on the steam bath and evaporate to dryness. Dissolve the resulting residue in 2 ml of concentrated hydrochloric acid and dilute the solution to a volume of 50 ml. Add 30 to 50 mg of crystalline ammonium persulfate and 3 ml of a 30-percent solution of ammonium thiocyanate. Compare the red color with that produced by known amounts of iron. Iron in an amount as small as 0.05 mg can be easily determined; this corresponds to 1 ppm in the sulfur.

### 4.5. Determination of Total Carbon

Organic impurities, reported as carbon, were determined by ascertaining the amount of carbon dioxide produced on burning a weighed portion of the sulfur. If the amount of carbon dioxide is large, more than 1 mg, it can be absorbed in a tared soda-lime tube and determined gravimetrically. If the amount is small, less than 1 mg, it can be absorbed in diluted ammonium hydroxide and determined turbidimetrically.

#### a. Apparatus

**Figure 2:**
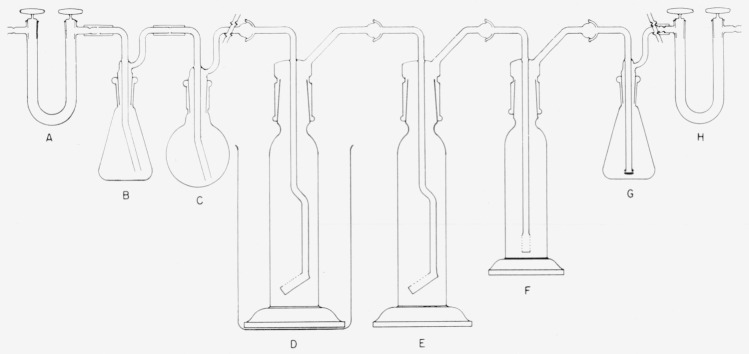
Apparatus used in determining carbon in sulfur.

The apparatus used for determining small amounts of carbon dioxide is shown in figure 2. The U-tube, A, contains soda-lime to remove carbon dioxide from the oxygen which is used for combustion. The 125-ml wash bottle, B, contains concentrated sulfuric acid to remove water. The 250-ml round-bottom flask, C, is fitted with a delivery tube which reaches to about 1 in. from the bottom of the flask. The sulfur is placed in this flask. Washing towers, D, E, and F, each contain 200 ml of 30-percent hydrogen peroxide to absorb the sulfur dioxide formed on combustion. Tower D is chilled in an ice bath. The 125-ml wash bottle, G, contains 50 ml of diluted ammonium hydroxide (1 + 9) to absorb the carbon dioxide which is formed in the combustion. The protective U-tube, H, contains soda-lime. Ball and socket joints are used for connections and all joints are lubricated with phosphoric acid.

If the amount of carbon dioxide to be determined is greater than 1 mg, flask G is replaced with a gas washing tower containing sulfuric acid, a U-tube containing magnesium perchlorate, and a tared U-tube containing soda-lime and magnesium perchlorate.

#### b. Recommended Procedure

##### (1) For Small Amounts of Carbon (less than 1 mg as *CO*_2_)

Assemble the apparatus shown in figure 2, but without the ammonia solution in G. Weigh between 75 and 100 g of sulfur and place it in flask C. Sweep out the entire apparatus with oxygen for at least 2 hr and then add the ammonia solution to flask G.

Regulate the stream of oxygen to a rate of about 10 ml per minute and heat the outside of flask C until the sulfur ignites. Continue burning until all the sulfur is consumed and then heat the bottom and lower sides of the flask to dull red heat. Continue passing the oxygen for at least ½ hr.

Remove flask G and add 5 ml of 10-percent barium chloride solution. Transfer the resulting solution to a Nessler tube and compare the turbidity to controls containing known amounts of carbonate in an equal volume of solution.

##### (2) For Larger Amounts of Carbon (more than 1 mg as *CO*_2_)

Assemble the apparatus shown in figure 2, but for flask *G*, substitute the tower containing sulfuric acid, the U-tube containing magnesium perchlorate, and the tared U-tube containing the soda-lime and magnesium perchlorate.

Follow the procedure outlined above for the addition and ignition of the sulfur.

Remove the tared U-tube containing the soda-lime and absorbed CO_2_ and weigh it. The amount of carbon dioxide is determined from the increase in weight of the soda-lime tube.

### 4.6. Determination of Sulfuric Acid

Sulfuric acid was determined by placing a known weight of sulfur in an ampoule with water, expelling the air with nitrogen, sealing the ampoule, melting the sulfur, and extracting the sulfuric acid into the water. The water is removed by decantation and the acid content is determined by titration with 0.02 *N* sodium hydroxide solution.

Repeated extractions failed to reduce the sulfuric acid content of the purified material below 0.0002 percent. However, since some or all of this acid may have been formed on the surface of the sulfur by atmospheric action or by a slow reaction with the water [8] the actual amount of sulfuric acid in the sulfur may have been less than the value obtained.

## 5. Analytical Results

Table 1 gives the results of the determination of the impurities in the sulfur taken as the starting material and in that obtained as the final product of purification.

## 6. Summary

Commercial roll sulfur was purified by a nitric acid—sulfuric acid oxidation method. The total impurities in this sulfur were reduced to 1.3×10^−5^ mole percent. The organic matter was reduced to 0.0002 percent as carbon.

## Figures and Tables

**Table 1:** Determination of impurities

Impurity	Amount of impurity in starting material	Amount of impurity in the purified product
		
	%	%
Total carbon	0.014	0.0002
Nonvolatile matter	.0076	.0003
Iron	.0011	.0001
Selenium	<.0001	(1)
Tellurium	<.00005	(1)
Arsenic	<.00005	(1)
Sulfuric acid	…………	.0002

1 Not determined.

## Footnotes

1 Figures in brackets indicate literature references at the end of this paper.

2 Throughout this paper dilutions are indicated by the volumes of concentrated acid and water mixed to prepare a diluted reagent. Diluted nitric (1+*x*) means a diluted acid prepared by mixing 1 volume of the concentrated acid with *x* volumes of water.
